# Associations between Drug Use and Sexual Risk Behaviours among Men Who Have Sex with Men in Japan: Results from the Cross-Sectional LASH Study

**DOI:** 10.3390/ijerph20136275

**Published:** 2023-07-02

**Authors:** Takeshi Miwa, Masazumi Yamaguchi, Tomoko Ohtsuki, Gaku Oshima, Chihiro Wakabayashi, Sachiko Nosaka, Kanna Hayashi, Yuzuru Ikushima, Masayoshi Tarui

**Affiliations:** 1 Positive Living and Community Empowerment Tokyo (PLACE TOKYO), 4-11-5-403 Takadanobaba, Shinjuku-ku, Tokyo 169-0075, Japan; 2 Hakujikai Choju Rehabilitation Hospital, 5-13-7 Shikahama, Adachi-ku, Tokyo 123-0864, Japan; 3 School of Information and Communication, Meiji University, 1-1 Kanda Surugadai, Chiyoda-ku, Tokyo 101-8301, Japan; 4 School of Health and Social Services, Saitama Prefectural University, 820 Sannomiya, Koshigaya-shi, Saitama 343-8540, Japan; 5 Graduate School of Human Sciences, Osaka University, 1-2 Yamadaoka, Suita-shi, Osaka 565-0871, Japan; 6 Faculty of Health Sciences, Simon Fraser University, Blusson Hall, Room 11300, 8888 University Drive, Burnaby, BC V5A 1S6, Canada

**Keywords:** drug use, sexual risk behaviour, men who have sex with men, HIV/AIDS, Japan

## Abstract

This study assessed drug use patterns among men who have sex with men (MSM) in Japan, and evaluated their potential associations with sexual risk behaviours. Between September and October 2016, study subjects were recruited through a cross-sectional survey (LASH: Love Life and Sexual Health) using a geosocial networking application for MSM. Of the participants, 25.4% (1756/6921) reported ever having used drugs, and 11.3% (780/6921) reported having done so in the past six months. Those who used drugs were more likely to have greater knowledge of HIV/sexually transmitted infections (STIs). Drug use in the past six months was independently associated with each of the following sexual risk behaviours in the same period: (i) six or more sexual partners (adjusted odds ratio [aOR] = 2.70, 95% confidence interval [CI]: 2.30–3.17); (ii) condomless anal intercourse (aOR = 2.88, 95% CI: 2.43–3.42); (iii) group sex (aOR = 2.60, 95% CI: 2.22–3.05); and (iv) sex work (aOR = 2.30, 95% CI: 1.67–3.16). These results suggest that MSM in Japan who use drugs are more likely to report sexual risk behaviours, while also having greater knowledge of HIV/STIs. Supporting MSM to minimise the harm from drug use may be helpful in reducing HIV transmission among this priority population.

## 1. Introduction

The use of drugs, encompassing licit and illicit substances, is an important and growing concern among men who have sex with men (MSM) in Japan. Although the prevalence of illicit drug use in Japan remains relatively low (<3.0%) among the general population [1,2], Nishijima et al. [3] documented that approximately 40% of HIV-positive MSM in Japan reported illicit drug use. The higher prevalence of drug use among MSM than that among the general population is consistent with findings from previous studies conducted in Western countries [4,5]. While MSM are believed to only account for less than 3% of the total male population in Japan [6,7], the high prevalence of drug use among MSM is concerning, as there are indications that drug use is associated with sexual risk behaviours [8], which could lead to an increase in sexually transmitted infections (STIs), including HIV.

Association between drug use and sexual risk behaviours is of particular concern, as MSM are already disproportionately affected by HIV infections worldwide [9]. In Japan, MSM accounted for more than 70% of new HIV infections between 2011 and 2015 [10], and HIV prevalence among MSM is expected to rise from about 3% [11] to 9.0% by 2050 if no new policy intervention is adopted [12]. Sexual risk behaviours in MSM include condomless anal intercourse (CLAI), which has a HIV transmission rate 18 times higher than that of vaginal intercourse [13]. Although the transmission of HIV through receptive CLAI can be effectively reduced by using condoms correctly [14], the consistent use of condoms among Japanese MSM remains a challenge. In an online self-administered survey conducted between 2016 and 2017 in Tokyo, 18% of MSM with casual male partners reported inconsistent condom use [15]. Previous studies have also documented that group sex engagement among MSM was associated with CLAI, as well as with illicit drug use [16,17]. In addition, sex work is another sexual behaviour that may increase the risk of HIV transmission among MSM, as sex work often involves uneven power dynamics during sex. A study conducted in Guatemala reported that MSM sex workers are at a higher risk of experiencing forced sex compared to other MSM [18], suggesting their vulnerability to HIV infection. 

As drug use may promote such sexual risk behaviours, and thus could exacerbate HIV infection rates among MSM, it is important to explore the current situation of MSM in Japan, to develop country-specific programmes catering to the needs of this priority population. However, the research on sexual behaviours and drug use among MSM in Japan is limited. Although a research on sexual behaviours and drug use among MSM in Japan was conducted between February and May 2003 [19], the patterns of drug use could have changed dramatically, as several restrictions on psychoactive substances have been enforced in Japan since then. In 2006, the Pharmaceutical Affairs Law in Japan established a new category called ‘Designated Substances’. The supply of psychoactive substances, which were classified into this category, including tryptamine-type derivatives (e.g., 5-methoxy-N, N-diisopropyltryptamine, or 5-MeO-DIPT) and alkyl nitrites, has been incrementally prohibited [20]. Subsequently, a new regulation enacted in 2014 prohibited the possession and consumption of these Designated Substances [21]. These drastic regulatory changes call for further research into the changing drug-use patterns among MSM, and their associations with sexual risk behaviours. 

Therefore, this study aimed to assess recent drug-use patterns among MSM in Japan, and evaluate their potential associations with HIV-related sexual risk behaviours. This study hypothesised that there would be some differences in psychosocial and clinical characteristics (e.g., age, HIV status, and mental health) between MSM in Japan who use drugs and those who do not, and that drug use is associated with certain sexual risk behaviours, with a particular focus on the number of sexual partners, and experience of CLAI, group sex, and sex work. Understanding the associations between drug use and sexual behaviours among this priority population would potentially help tailor HIV-prevention interventions for MSM who use drugs.

## 2. Materials and Methods

Data were derived from a cross-sectional online behavioural survey named Love Life and Sexual Health (LASH), which was conducted between September and October 2016. A banner advertising the study was posted on a Japanese geosocial networking application commonly used by gay and bisexual men in Japan. Users of the application who clicked on the banner were guided to a website that explained the purpose of the study and the voluntary participation, including participants’ ability to leave the survey before completing all the questions. Informed consent was requested on this page, and only those who agreed to participate were given access to the questionnaire. While the survey ensured complete anonymity, the cookie data were collected to prevent duplicate responses. The study protocol was approved by the Ethics Committee of PLACE TOKYO (2015-P01).

Since this study recruited subjects solely through a gay-specific geosocial networking application, those who agreed to participate were all assumed to be MSM. However, consenting respondents were first asked about their gender identity, and those who were “genetically female and self-identified as female” were deemed ineligible for the study. In this study, men were defined by sex at birth as well as by current gender identity, which meant that respondents who self-identified as transgender men or women were both included as MSM. As the gay-specific geosocial networking application in Japan prohibits use by those under 18 years old, this study did not expect answers from minors. However, the study did not particularly exclude respondents based on age during the analysis stage, in order to reflect the actual situation of application users. A total of 28 respondents reported that they were under 18 years old. 

The questionnaire items included information on socio-demographics (e.g., age, educational background, gender identity, sexual orientation, etc.), sexual behaviours, HIV status, HIV/STI knowledge, experience of drug use, and mental health. Respondents were asked about the following sexual behaviours in the past six months: (i) number of sexual partners; (ii) CLAI; (iii) group sex (having sex with multiple partners at the same time); and (iv) sex work (receiving money in exchange for sex). Drugs used in the past six months included: erectile dysfunction drugs, codeine-containing cough medicines, new psychoactive substances (e.g., synthetic cannabinoids, synthetic cathinones, phenethylamine and thiophene derivatives), 5-MeO-DIPT (5-methoxy-N,N-diisopropyltryptamine), alkyl nitrites (rush/poppers), inhalants (aerosol/gas), thinner, marijuana, methamphetamine, MDMA (3,4-Methylenedioxymethamphetamine), heroin, cocaine, ketamine, and GHB (gamma-Hydroxybutyric acid). For new psychoactive substances, we used the term “quasi-legal drugs (herbs, liquid, powder, aroma, salt)” in the questionnaire, because it is commonly used to refer to the aforementioned substances in Japan. The questionnaire also asked whether they injected drugs.

The questionnaire also assessed the mental health of the respondents using the Japanese version of the Kessler 6-Item Psychological Distress scale (K6 scale) [22]. Respondents were asked to self-report how frequently they felt the following symptoms in the past month: (i) felt nervous; (ii) felt hopeless; (iii) felt restless or fidgety; (iii) felt depressed; (iv) felt that everything was an effort; and (v) felt worthless. A value between zero and four were assigned to each response ranging from “None of the time” to “All of the time” [23].

HIV/STI knowledge was assessed using ten true-or-false items, originally developed for this research: (i) “Even if you are HIV-positive, the virus becomes almost undetectable in the blood if you are under continuous treatment”; (ii) “You become more vulnerable to HIV infection if you are infected with other STIs”; (iii) “Even if you are infected with HIV, starting treatment at an early stage will enable you to live a long life”; (iv) “There are social benefits to minimise the cost of HIV treatment”; (v) “Even if you are under HIV treatment, your personal information will be protected and will not be disclosed to your workplace/school without your consent”; (vi) “Even if you are HIV-positive, the risk of HIV transmission is negligible if you are aware of your status and under appropriate treatment”; (vii) “HIV can be transmitted to your sexual partner if you are HIV-positive and unaware of it”; (viii) “The risk of HIV transmission through oral sex is low, but not zero”; (ix) “Most HIV infections in developed countries occur through male gay sex”; and (x) “The use of condoms lowers the risk of transmitting not only HIV but also other STIs.” A value of one was assigned to each correct answer (“Yes”), and a value of zero was assigned to each wrong answer (“No”). Respondents were scored between 0 and 10 based on the number of correct answers. A median was used as a cut-off point to divide the respondents into two groups: those with greater HIV/STI knowledge, and those with lesser HIV/STI knowledge.

Among the 10,544 who agreed to participate in the study, the following respondents were excluded through data cleaning: (i) 979 who did not answer questions on basic socio-demographic information, (ii) 25 who answered the questionnaire twice (identified by cookie data), and (iii) 820 who had inconsistent answers (e.g., “graduated from university” but “younger than 16 years old”). Among the remaining 8720 respondents, 6921 with complete answers were used for data analyses.

Simple descriptive statistics (proportions, means, and 95% confidence intervals [CIs]) were used to describe the respondent characteristics. As participation in the study was voluntary, the samples were not completely random or independent; thus, bootstrapping was employed to derive CIs [24]. The characteristics of those who used drugs and those who did not were compared using t-tests (Welch’s/Student’s), and Pearson’s chi-squared tests.

In order to match the time frame of drug use and sexual behaviours (i.e., past six months), respondents who had used any of the aforementioned drugs in the past six months were categorised as “Those who used drugs”, while the rest were categorised as “Those who did not use drugs”, unless stated otherwise. Simple and multivariable logistic regression analyses were conducted to explore the associations between drug use (independent variable) and each sexual behaviour (dependent variable) in the past six months. The control variables in the regression model were age, university education, HIV status, and HIV/STI knowledge, as a previous study suggested associations of these factors with drug use [19]. All statistical analyses were performed using R (version 4.1.3). All *p*-values were two-sided, and the significance level adopted was 0.05.

## 3. Results

### 3.1. Overall Respondent Characteristics

Among the eligible 6921 respondents, the median age was 33 years (interquartile range [IQR]: 26–41) (see Table 1). Five samples were excluded when estimating the mean and median of age, as their data were qualitative (i.e., “Under 16 years old” (*n* = 4) and “81 years old and above” (*n* = 1)). A total of 98.0% (6782/6921) were Japanese nationals, and 53.2% (3679/6921) had university degrees or were currently university students. Most respondents self-identified as male and homosexual/bisexual. 0.3% (23/6921) self-identified as transgender men, and 0.6% (41/6921) as transgender women. A total of 15.7% (1089/6921) had a K6 score higher than or equal to 13, which is a cut-off point for serious mental illness (see Table 2) [22]. A total of 2609 respondents had no lifetime experience of HIV testing, and 32 respondents had taken the HIV test but had not checked the results; thus, the percentage of those who were unaware of their HIV status was 38.2% ((2609 + 32)/6921). 

### 3.2. Characteristics of Those Who Used Drugs

The median age of those who used drugs was 39 years (IQR:31–44), whereas it was 33 years (IQR: 25–41) for those who did not (see Table 1). Those who used drugs were more likely to self-identify as homosexual (gay), and drink alcohol almost every day during the past six months (see Table 2). While 21.5% (168/780) of those who used drugs were HIV-positive compared with 5.6% (345/6141) of those who did not, the percentage of those who were unaware of their HIV status was significantly lower among those who used drugs (16.2%, 126/780) than among those who did not (41.0%, 2515/6141). Regarding HIV/STI knowledge, the number of participants who scored higher than or equal to the median (i.e., 8) was more concentrated among those who used drugs (73.3%, 572/780) than among those who did not (58.4%, 3588/6141). However, there was no statistically significant difference in the K6 scores between the two groups (*p* = 0.240).

### 3.3. Sexual Behaviours of Respondents

As shown in Table 3, 35.8% (2478/6921) had six or more male sexual partners in the past six months. The majority of respondents had between two and five male sexual partners, while 8.9% (613/6921) reported having no male sexual partner in the past six months. About half of the respondents (48.6%, 3364/6921) had CLAI, and 30.7% (2126/6921) had experienced group sex in the past six months. The proportion of those who had engaged in sex work was 4.1% (285/6921). While the majority of respondents were sexually active, their lifetime experience with HIV testing was 62.3% (4312/6921).

### 3.4. Types of Drugs Used

**Figure 1:**
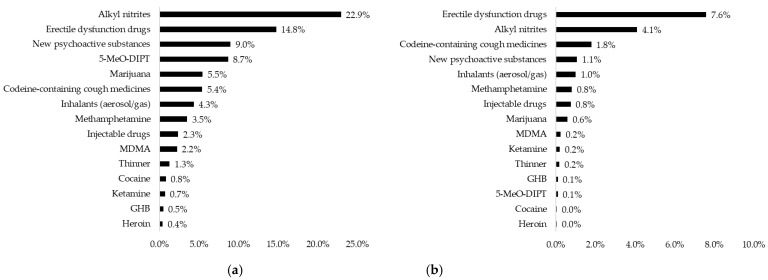
Drug use among 6921 individuals. (**a**) Prevalence of lifetime experience of drug use; (**b**) prevalence of drug use in the past six months; 5-MeO-DIPT, 5-methoxy-N,N-diisopropyltryptamine; MDMA, 3,4-Methylenedioxymethamphetamine; GHB, gamma-Hydroxybutyric acid.

Of all respondents, 25.4% (1756/6921) reported that they had used drugs some time in their life, and 11.3% (780/6921) reported having done so in the past six months (see Table 2). Figure 1 shows the prevalence of drug use among the 6921 respondents. Regarding lifetime experience, the most commonly used drugs were alkyl nitrites (22.9%, 1587/6921), erectile dysfunction drugs (14.8%, 1022/6921), and new psychoactive substances (9.0%, 622/6921). In contrast, the most commonly used drugs in the past six months were erectile dysfunction drugs (7.6%, 526/6921), followed by alkyl nitrites (4.1%, 284/6921), and codeine-containing cough medicines (1.8%, 124/6921). The prevalence of drug use in the past six months was lower than 1% for the ten drugs included in the questionnaire. For example, while 8.7% (600/6921) of respondents had a lifetime experience of using 5-MeO-DIPT, only 0.1% (7/6921) had used it in the past six months.

### 3.5. Associations between Drug Use and Sexual Behaviours

As shown in Table 4, multivariable logistic regression analyses showed that drug use in the past six months was independently associated with each of the following sexual risk behaviours in the same period: (i) six or more sexual partners (adjusted odds ratio [aOR] = 2.70, 95% confidence interval [CI]: 2.30–3.17); (ii) CLAI (aOR = 2.88, 95% CI: 2.43–3.42); (iii) group sex (aOR = 2.60, 95% CI: 2.22–3.05); and (iv) sex work (aOR = 2.30, 95% CI: 1.67–3.16).

## 4. Discussion

### 4.1. Characteristics of Those Who Used Drugs

The findings of this study corroborate the results of the previous study about those who use drugs being more likely to be older and self-identify as gay [19]. The reason why those who used drugs were slightly older could be partly explained by the inclusion of erectile dysfunction drugs and alkyl nitrites in this study. While some young men are also affected by erectile dysfunction, the incidence is generally known to increase with age [25]. The older generation may also be less hesitant to use alkyl nitrites compared to the younger generation, who were likely to have been exposed to the gay community after the supply of this substance was prohibited in Japan. In addition, those who clearly self-identify as “gay” may be more attached, compared to bisexual and questioning men, to the gay community, which is reported to be associated with drug-related problems [26].

On the other hand, this study did not identify any significant difference in probable psychological distress between those who used drugs and those who did not. A previous study [27] also documented that the K6 scale was not associated with new psychoactive substance use, despite some studies indicating that those among the general population who use drugs have high rates of mental health disorders, including depression and anxiety [28,29].

The null association between drug use and mental health in this study may be partly explained by the presence of the gay community in Japan that uses drugs. A cross-sectional internet-based survey that recruited MSM from twelve different Asian countries found that MSM who use stimulant drugs are likely to have more gay friends compared to non-users [30]. Therefore, one hypothesis is that those who use drugs could experience a stronger sense of belonging to the MSM community through drug use, thus making the subjective perception of their own wellbeing more positive. Another likely hypothesis is that the K6 scale cannot capture mild mental distress [31], which, compared to serious mental illnesses, may be more prevalent among MSM who use drugs. In addition, MSM in this study may have used drugs mostly for recreational purposes in sexual contexts, and thus, the study could not identify mental health problems that are more concerning among people who are dependent on drugs. Further research is needed to explore the motivations for drug use and the severity of drug dependence, and their associations with mental health problems.

### 4.2. Associations between Drug Use and Sexual Risk Behaviours

More importantly, this study suggests that MSM who use drugs, despite having a greater knowledge of HIV/STIs, are more likely to engage in sexual risk behaviours, particularly CLAI. The mechanism underlying the association between drug use and CLAI is often complex, and involves several psychological and social factors. One possible explanation is the high-risk hypothesis, in which people who use drugs tend to live riskier lives [32], and thus increase their likelihood of engaging in risky sex. In other words, drug use and CLAI may both be influenced by a similar personality trait that impacts decision-making processes. In a study conducted in Israel, those with drug-use disorders were reported to share similar personality profiles with those with compulsive sexual behaviour [33]. 

Another possibility is that some MSM use drugs and practise CLAI to relieve emotional pain and stress, as suggested in the self-medication hypothesis [34] and a study conducted by Folkman et al. [35]. This hypothesis may partly explain why those who used drugs in this study were well-informed about safer sex, but were more likely to report CLAI. The high proportion of HIV testing experience among MSM who used drugs also suggests that they are aware of the risk in CLAI. Hence, for some MSM, using drugs and engaging in CLAI may temporarily relieve stress, and bring pleasure to themselves and their sexual partners, which may be a higher priority compared to utilising their knowledge to protect their own health.

Similarly, the association between drug use and sex work may be explained in part by the aforementioned hypotheses. While previous studies conducted in the Philippines and Iran suggest that some people who use drugs turn to sex work to purchase drugs [36,37], the related research for the MSM population is too limited to explore this scenario in Japan. Further research, both quantitative and qualitative, is needed to better understand the psychological factors that promote or mediate drug use and sexual risk behaviours among MSM, including CLAI and sex work. Possible factors include the experience of discrimination, having internalised homophobia, or having high gay-related rejection sensitivity, as implicated in previous studies [26,38,39,40].

In regards to other sexual risk behaviours, there may be complex associations between drug use, group sex, the number of sexual partners, and CLAI. The increased risk of HIV infection from group sex per se remains contentious, as a previous study documented that condom use was more often reported during group sex, compared to dyadic sex [41]. However, it is important to note that “group sex events” often involve drug use and a high turnover of sexual partners. In fact, there were indications that attendees of such events were more likely to report drug use and CLAI [42]. Those who used drugs at group sex events were also more likely than those who did not to report CLAI [43]. Therefore, this study implies the possibility that using drugs during group sex events is not uncommon among the MSM community in Japan. Frequent drug use during group sex events may promote CLAI with multiple sexual partners, and eventually pose the risk of HIV transmission.

Nonetheless, the fact that MSM in Japan who use drugs tend to practise sexual risk behaviours, albeit being well-informed about safer sex, provides an important insight into the limitations of current HIV-prevention programmes in Japan, which often focus solely on delivering information. Furthermore, current abstinence-orientated interventions in Japan for those who use drugs may overlook the barriers to safer sex practices upon drug use. To minimise HIV transmission among MSM who use drugs, peer-led programmes that reflect the idea of harm reduction may be helpful. Examples of such programmes include: (i) assertiveness training to help participants practise how to express their own opinions in sexual contexts; (ii) self-awareness groups to help participants understand their own feelings, thoughts, and personality patterns that influence sexual behavioural tendencies; and (iii) self-help groups for MSM to talk about their drug use and sexual experiences in a safe environment. Further research is needed, to assess the effectiveness of such personalised interventions and social support services that are tailored to the needs of this priority population.

### 4.3. Limitations and Strengths

The results of this study should be interpreted in light of several limitations. Firstly, this study recruited subjects from only one Japanese geosocial networking application, and thus may not necessarily represent the situation of MSM in Japan or elsewhere. MSM who do not use the geosocial networking application, MSM who are not accustomed to internet-based surveys, and those who cannot read Japanese (including foreigners), were most likely unable to participate in the survey. Secondly, there may be several reporting biases, especially recall bias related to drug use and sexual behaviours, though the complete anonymity of the questionnaire may have minimised the occurrence of socially desirable responses. In addition, although respondents were free to leave the survey whenever they wanted, the system was designed to encourage complete answers, which may have affected the reliability of some answers. Finally, owing to the cross-sectional design, we could not infer causation from the associations observed in this study.

On the other hand, this study was designed to increase the diversity of respondents. Firstly, the use of a nationwide geosocial networking application allowed the study to collect large samples not only from the metropolitan area of Tokyo, but also from all other prefectures in Japan. Using the geosocial networking application also ensured the inclusion to a certain extent of MSM who were not attached to the gay community, which is estimated to make up about two-thirds of Japanese MSM [44]. These MSM are often not easily accessible through gay-venue-based surveys [45]. Furthermore, this study used popular gay models on advertisements, and encouraged responses to “deepen self-understanding about your own romantic and sexual relationships”. This approach possibly allowed the study to also reach those among the MSM population who are usually less interested in the topics of sexual and mental health.

## 5. Conclusions

This study, which utilised a Japanese geosocial networking application to recruit subjects, found that MSM in Japan who used drugs were more likely than those who did not to report sexual risk behaviours, while also having greater knowledge of HIV/STIs. Needless to say, disseminating information to the MSM community about the risks of drug use and the importance of safer sex is vital. However, it may also be important to develop more harm-reduction programmes that are catered to the needs and preferences of MSM who use drugs, such as community-led workshops to help them understand their own communication patterns that influence their sexual behaviours, and self-help groups to discuss their drug use and sexual experience with peers.

## Figures and Tables

**Table 1:** Socio-demographics of respondents.

Characteristic	Total (*n* = 6921)			Those Who Did Not Use Drugs (*n* = 6141)			Those Who Used Drugs ^a^ (*n* = 780)			Statistics	*p*-Value ^b^
	*n*	%	95% CI	*n*	%	95% CI	*n*	%	95% CI		
Age											
Mean	33.8	(95% CI: 33.6–34.0)		33.3	(95% CI: 33.1–33.5)		37.8	(95% CI: 37.1–38.4)		13.398	<0.001
Median	33	(IQR: 26–41)		33	(IQR: 25–41)		39	(IQR: 31–44)			
<19	111	1.6%	(1.3–1.9)	107	1.7%	(1.4–2.0)	4	0.5%	(0.1–1.0)	131.936	<0.001
19–34	3722	53.8%	(52.6–55.0)	3441	56.0%	(54.8–57.2)	281	36.0%	(32.7–39.4)		
35–49	2695	38.9%	(37.8–40.2)	2273	37.0%	(35.8–38.2)	422	54.1%	(50.6–57.4)		
50–64	382	5.5%	(5.0–6.1)	310	5.0%	(4.4–5.6)	72	9.2%	(7.2–11.2)		
65+	11	0.2%	(0.1–0.3)	10	0.2%	(0.1–0.3)	1	0.1%	(0.0–0.4)		
Nationality											
Japanese	6782	98.0%	(97.6–98.3)	6019	98.0%	(97.7–98.4)	763	97.8%	(96.7–98.7)	0.131	0.718
non-Japanese	139	2.0%	(1.7–2.4)	122	2.0%	(1.7–2.3)	17	2.2%	(1.3–3.2)		
University Education											
No	3242	46.8%	(45.6–48.0)	2822	46.0%	(44.7–47.1)	420	53.8%	(50.6–57.4)	17.315	<0.001
Yes	3679	53.2%	(51.9–54.3)	3319	54.0%	(52.8–55.3)	360	46.2%	(42.7–49.9)		
Gender Identity											
Male	6824	98.6%	(98.3–98.6)	6051	98.5%	(98.2–98.8)	773	99.1%	(98.3–99.7)	2.007	0.553
Transgender man	23	0.3%	(0.2–0.5)	21	0.3%	(0.2–0.5)	2	0.3%	(0.0–0.6)		
Transgender woman	41	0.6%	(0.4–0.8)	39	0.6%	(0.4–0.8)	2	0.3%	(0.0–0.6)		
Others	33	0.5%	(0.3–0.7)	30	0.5%	(0.3–0.7)	3	0.4%	(0.0–0.9)		
Sexual orientation											
Homosexual	5503	79.5%	(78.6–80.4)	4820	78.5%	(77.5–79.5)	683	87.6%	(85.3–89.9)	36.993	0.001
Bisexual	1126	16.3%	(15.4–17.2)	1046	17.0%	(16.1–17.9)	80	10.3%	(8.2–12.3)		
Heterosexual	21	0.3%	(0.2–0.4)	18	0.3%	(0.2–0.4)	3	0.4%	(0.0–0.9)		
Others	271	3.9%	(3.5–4.4)	257	4.2%	(3.7–4.7)	14	1.8%	(0.9–2.7)		

^a^ Drug use in the past six months. ^b^ Monte Carlo simulation is computed when applicable. *p*-values smaller than 0.001 are all shown as <0.001. CI, confidence interval. IQR, interquartile range.

**Table 2:** Clinical characteristics of respondents.

Characteristic	Total (*n* = 6921)			Those Who Did Not Use Drugs (*n* = 6141)			Those Who Used Drugs ^a^ (*n* = 780)			Statistics	*p*-Value ^b^
	*n*	%	95% CI	*n*	%	95% CI	*n*	%	95% CI		
HIV status											
Negative	3767	54.4%	(53.2–55.6)	3281	53.4%	(52.1–54.6)	486	62.3%	(58.6–65.8)	358.242	<0.001
Unknown	2641	38.2%	(37.0–39.4)	2515	41.0%	(39.8–42.1)	126	16.2%	(13.8–18.7)		
Positive	513	7.4%	(6.8–8.0)	345	5.6%	(5.0–6.2)	168	21.5%	(18.6–24.2)		
HIV/STI knowledge											
Mean	7.8	(95% CI: 7.8–7.9)		7.8	(95% CI: 7.7–7.8)		8.4	(95% CI: 8.3–8.5)		10.895	<0.001
Median	8	(IQR: 7–9)		8	(IQR: 7–9)		9	(IQR: 7–10)			
Low (0–7)	2761	39.9%	(38.8–41.0)	2553	41.6%	(40.4–42.7)	208	26.7%	(23.6–29.9)	64.134	<0.001
High (8+)	4160	60.1%	(59.0–61.3)	3588	58.4%	(57.2–59.6)	572	73.3%	(70.3–76.5)		
K6 score											
Mean	6.6	(95% CI: 6.4–6.7)		6.6	(95% CI: 6.4–6.7)		6.8	(95% CI: 6.4–7.3)		1.176	0.240
Median	5	(IQR: 2–10)		5	(IQR: 2–10)		6	(IQR: 2–10)			
Low (0–12)	5832	84.3%	(83.4–85.0)	5187	84.5%	(83.6–85.4)	645	82.7%	(80.0–85.3)	1.640	0.200
High (13+)	1089	15.7%	(14.9–16.6)	954	15.5%	(14.7–16.5)	135	17.3%	(14.7–20.1)		
Alcohol-drinking habit in the past six months											
Almost every day	1089	15.7%	(14.8–16.6)	907	14.8%	(13.9–15.6)	182	23.3%	(20.6–26.2)	38.480	<0.001
Sometimes	4807	69.5%	(68.4–70.5)	4310	70.2%	(69.0–71.3)	497	63.7%	(60.3–67.2)		
Not at all	1025	14.8%	(14.0–15.6)	924	15.0%	(14.2–16.0)	101	12.9%	(10.5–15.5)		
Lifetime experience of drug use											
No	5165	74.6%	(73.6–75.7)								
Yes	1756	25.4%	(24.4–26.4)								
Experience of drug use in the past six months											
No	6141	88.7%	(87.9–89.5)								
Yes	780	11.3%	(10.6–12.0)								

^a^ Drug use in the past six months. ^b^ *p*-values smaller than 0.001 are all shown as <0.001. STI, sexually transmitted infection. CI, confidence interval. IQR, interquartile range.

**Table 3:** Sexual behaviours of respondents.

Behaviour	Total (*n* = 6921)			Those Who Did Not Use Drugs (*n* = 6141)			Those Who Used Drugs ^a^ (*n* = 780)			Statistics	*p*-Value ^b^
	*n*	%	95% CI	*n*	%	95% CI	*n*	%	95% CI		
No. of male sexual partners in the past six months											
0	613	8.9%	(8.2–9.6)	603	9.8%	(9.0–10.6)	10	1.3%	(0.6–2.2)	335.508	<0.001
1	815	11.8%	(11.0–12.6)	778	12.7%	(11.8–13.5)	37	4.7%	(3.3–6.5)		
2–5	3015	43.6%	(42.3–44.8)	2753	44.8%	(43.6–46.0)	262	33.6%	(30.3–37.1)		
6–10	1178	17.0%	(16.1–17.9)	1001	16.3%	(15.4–17.2)	177	22.7%	(19.5–25.8)		
11–20	708	10.2%	(9.6–10.9)	577	9.4%	(8.7–10.1)	131	16.8%	(14.1–19.5)		
21–50	417	6.0%	(5.5–6.6)	314	5.1%	(4.6–5.7)	103	13.2%	(10.8–15.6)		
51+	175	2.5%	(2.2–2.9)	115	1.9%	(1.5–2.2)	60	7.7%	(5.8–9.6)		
Experience of CLAI in the past six months											
No	3557	51.4%	(50.2–52.5)	3346	54.5%	(53.2–55.7)	211	27.1%	(23.8–30.1)	208.531	<0.001
Yes	3364	48.6%	(47.4–49.8)	2795	45.5%	(44.3–46.7)	569	72.9%	(69.9–75.9)		
Experience of group sex in the past six months											
No	4795	69.3%	(68.2–70.3)	4443	72.3%	(71.2–73.5)	352	45.1%	(41.5–48.6)	240.978	<0.001
Yes	2126	30.7%	(29.7–31.8)	1698	27.7%	(26.6–28.8)	428	54.9%	(51.4–58.2)		
Experience of sex work in the past six months											
No	6636	95.9%	(95.4–96.4)	5915	96.3%	(95.9–96.8)	721	92.4%	(90.6–94.1)	26.442	<0.001
Yes	285	4.1%	(3.6–4.6)	226	3.7%	(3.2–4.1)	59	7.6%	(5.9–9.6)		
Ever tested for HIV											
No	2609	37.7%	(36.5–38.8)	2489	40.5%	(39.3–41.7)	120	15.4%	(12.8–18.2)	186.336	<0.001
Yes	4312	62.3%	(61.2–63.5)	3652	59.5%	(58.3–60.7)	660	84.6%	(81.9–87.1)		

^a^ Drug use in the past six months. ^b^ *p*-values smaller than 0.001 are all shown as <0.001. CI, confidence interval. CLAI, condomless anal intercourse.

**Table 4:** Associations of drug use in the past six months with sexual behaviours.

Sexual Behaviours in the Past Six Months	Drug Use ^b^		Prevalence (%)	Simple			Multivariable ^a^		
				OR	95% CI	*p*-Value	aOR	95% CI	*p*-Value
Six or more sexual partners	No	2007/6141	32.70%	Ref			Ref		
(0: No, 1: Yes)	Yes	471/780	60.40%	3.14	(2.69–3.66)	<0.001	2.70	(2.30–3.17)	<0.001
Experience of CLAI	No	2795/6141	45.50%	Ref			Ref		
(0: No, 1: Yes)	Yes	569/780	72.90%	3.23	(2.74–3.81)	<0.001	2.88	(2.43–3.42)	<0.001
Experience of group sex	No	1698/6141	27.70%	Ref			Ref		
(0: No, 1: Yes)	Yes	428/780	54.90%	3.18	(2.73–3.70)	<0.001	2.60	(2.22–3.05)	<0.001
Experience of sex work	No	226/6141	3.70%	Ref			Ref		
(0: No, 1: Yes)	Yes	59/780	7.60%	2.14	(1.59–2.88)	<0.001	2.30	(1.67–3.16)	<0.001

^a^ Adjusted for age, university education, HIV status, and HIV/STI knowledge. ^b^ Drug use in the past six months. CI, Confidence interval. aOR, adjusted odds ratio. Ref, reference. CLAI, condomless anal intercourse.

## Data Availability

The data that support this study cannot be publicly shared due to privacy reasons, but may be shared upon reasonable request to the corresponding author, if appropriate.
